# Targeted Serum Metabolite Profiling Identifies Metabolic Signatures in Patients with Alzheimer's Disease, Normal Pressure Hydrocephalus and Brain Tumor

**DOI:** 10.3389/fnins.2017.00747

**Published:** 2018-01-09

**Authors:** Matej Orešič, Gabriella Anderson, Ismo Mattila, Manoucher Manoucheri, Hilkka Soininen, Tuulia Hyötyläinen, Cherlynn Basignani

**Affiliations:** ^1^Turku Centre for Biotechnology, University of Turku and Åbo Akademi University, Turku, Finland; ^2^School of Medical Sciences, Örebro University, Örebro, Sweden; ^3^Florida Hospital Orlando, Neuroscience Research Institute, Orlando, FL, United States; ^4^Steno Diabetes Center Copenhagen, Gentofte, Denmark; ^5^Department of Neurology, Neuro Center, Kuopio University Hospital, Kuopio, Finland; ^6^Department of Neurology, Institute of Clinical Medicine, University of Eastern Finland, Kuopio, Finland; ^7^Department of Chemistry, Örebro University, Örebro, Sweden

**Keywords:** Alzheimer's disease, brain tumor, hypoxia, metabolomics, normal pressure hydrocephalus

## Abstract

Progression to AD is preceded by elevated levels of 2,4-dihydroxybutanoic acid (2,4-DHB), implicating hypoxia in early pathogenesis. Since hypoxia may play a role in multiple CNS disorders, we investigated serum metabolite profiles across three disorders, AD, Normal Pressure Hydrocephalus (NPH) and brain tumors (BT). Blood samples were collected from 27 NPH and 20 BT patients. The profiles of 21 metabolites were examined. Additionally, data from 37 AD patients and 46 controls from a previous study were analyzed together with the newly acquired data. No differences in 2,4-DHB were found across AD, NPH and BT samples. In the BT group, the fatty acids were increased as compared to HC and NPH groups, while the ketone body 3-hydroxybutyrate was increased as compared to AD. Glutamic acid was increased in AD as compared to the HC group. In the AD group, 3-hydroxybutyrate tended to be decreased with respect to all other groups (mean values −30% or more), but the differences were not statistically significant. Serine was increased in NPH as compared to BT. In conclusion, AD, NPH and BT have different metabolic profiles. This preliminary study may help in identifying the blood based markers that are specific to these three CNS diseases.

## Introduction

Recent studies have suggested that the development of Central Nervous System (CNS) disorders could be traced to alterations in metabolic pathway (Kaddurah-Daouk and Krishnan, 2009). Many diseases disturb metabolism processes, resulting in changes that can be captured as metabolic signatures. In a diseased state, the concentration of these metabolites are modified, providing information regarding disease pathophysiology.

Metabolomics is the study of the metabolome, the collection of small molecules found in cells and tissues (Kristal and Shurubor, 2005; Kaddurah-Daouk and Krishnan, 2009). Through metabolomics tools, quantitative data on a wide range of metabolites is analyzed to gain a more comprehensive understanding of how those metabolomics profiles are associated with different diseases (van der Greef et al., 2003). Therefore, metabolic signatures for CNS disorders could be key to identifying biomarkers for these diseases.

Cerebral ischemia, which occurs when there is insufficient blood flow to fulfill the brain's metabolic need, is a common characteristic among CNS disorders. Cerebral ischemia often leads to cerebral hypoxia, or limited oxygen supply in the brain. Three distinct CNS disorders which have been linked to cerebral hypoxia are Alzheimer's disease (AD), Normal Pressure Hydrocephalus (NPH), and brain tumors (BT) (Zagzag et al., 2000; Dombrowski et al., 2008; Daulatzai, 2013).

AD (a neurodegenerative disorder associated with the accumulation of extracellular amyloid-beta peptide in the brain), NPH (a communicating hydrocephalus characterized by the disruption of cerebrospinal fluid (CSF) flow regulation in the brain without an increase in intracranial pressure), and BT are vastly different diseases. However, they share similar symptoms such as dementia and urinary incontinence. These similarities can lead to misdiagnosis, particularly between AD and NPH. The commonalities among these three diseases will allow the identification of specific, reliable biomarkers that are reflective of disease associated biological processes.

In a previous study, we have shown that progression to AD is preceded by elevated levels of 2,4-dihydroxybutanoic acid (2,4-DHB; also known as 2,4-dihydroxybutyrate or 3-deoxytetronate), however, no significant changes were observed in the diagnosed AD with respect to this metabolite (Oresic et al., 2011). Although the underlying mechanisms and pathogenic relevance of elevated 2,4-DHB are not known, it has been recently suggested that the production of 2,4-DHB is linked to the hypoxia-induced activation of γ-aminobutyric acid (GABA) shunt and the conversion of GABA to γ-hydroxybutyrate, which can be subsequently metabolized to 2,4-DHB (Salminen et al., 2016). Multiple metabolomics studies have been conducted in BT patients (Griffin and Kauppinen, 2007). These studies together point to altered metabolic state in tumors, which is characterized by hypoxia (Jensen, 2009) and aerobic glycolysis (Warburg effect). No studies so far have systematically investigated metabolic changes in NPH.

Since hypoxia has been implicated in multiple Central Nervous System (CNS) disorders including AD, BT and NPH, here we aim to systematically investigate serum metabolite profiles across three CNS disorders, AD, NPH and BT, specifically gliomas, and thus identify potential similarities or specific differences.

## Materials and methods

### Study design

This prospective clinical study was approved by the Florida Hospital Institutional Review Board. All study subjects or legally authorized representatives provided written consent for study participation, which included biological sample collection and brief demographic data collection. All methods were performed in accordance with the relevant guidelines and regulations of this Review Board.

From July 2013 through July 2014, NPH participants were recruited from Florida Hospital's NPH program. The goal of the NPH program is to confirm the diagnosis of NPH and to select patients that would benefit from a permanent VPS. NPH participants met the following inclusion criteria: (a) Age ≥ 60 years, (b) Participation in the NPH program, and (c) Individuals willing and able to sign the informed consent either by themselves or with the assistance of an interpreter or legally authorized representative. Twenty-seven (27) NPH participants provided samples for this study. One participant was not included in the metabolomics analysis due to sample quality issues. In addition to the sample provided, participants' age, gender, and Mini Mental Status Examination (MMSE) were collected. Two NPH patients did not complete the MMSE. Their scores are included in the demographic statistics, up until they discontinued the testing.

From August 2014 to September 2014, participants diagnosed with BT were recruited from Florida Hospital's outpatient Neuro-Oncology office. All BT participants met the following inclusion criteria: (a) Age ≥ 60 years, (b) Diagnosed with gliomas and patient of Florida Hospital's Neuro-Oncology practice, and (c) Individuals willing and able to sign the informed consent either by themselves or with the assistance of an interpreter or legally authorized representative. Twenty (20) BT participants provided samples for this study. In addition to the sample provided, participants' age, gender, and tumor type were collected.

Ten (10) ml of blood serum was collected from each of the 27 NPH and 20 BT participants from a routine blood draw during their hospital stay or office visit. The samples were collected from non-fasting patients and there was no bias in the time of serum collection. Samples were collected in accordance to the schedule of the patients' hospital stay or office visit.

Each specimen vial was labeled with patient study number, date and time of collection, and type of biological sample. Within an hour of the collection, the samples were taken to Florida Hospital Pathology Laboratory, where they were processed and stored before shipment to the metabolomics laboratory.

Each blood sample was set to clot in an upright position for 30 min to 1 h before centrifuge. Each specimen was centrifuged for at least 15 min at 2,200–2,500 RPM within 1 h of collection. Specimens were set to freeze at −80 degrees Celsius. Specimen tubes were placed in freezer at a 45 degree angle to avoid tube breakage caused by expansion during freezing. Specimens were stored in the freezer at the Florida Hospital Pathology Laboratory.

Selected metabolomics data from 37 AD participants and 46 healthy control (HC) participants acquired from a previous study (Oresic et al., 2011) were included in the present study. In that study, blood samples were taken during morning hours and after fasting in most cases, and serum samples were analyzed by metabolomics.

### Metabolomic analysis

Serum samples (30 μl) were spiked with 10 μl of internal standard mixture (d4-succinic acid at concentration of 58.54 μg/ml, d5-glutamic acid – 110.43 μg/ml, d8-valine – 35.72 μg/ml, and heptadecanoic acid −175.36 μg/ml.; Sigma Aldrich). The protein precipitation was done as follows: 400 μl of methanol was added and then the samples were vortexed for 2 min. After centrifugation (3 min @ 10000 RPM) and settling for 30 min the supernatant was evaporated to dryness under gentle flow of nitrogen before derivatization. The original metabolites were then converted into their methoxime (MEOX) and trimethylsilyl (TMS) derivative(s) by two-step derivatization. First, 25 μL of methoxamine hydrochloride was added to the residue, and the mixture was incubated for 60 min at 45°C. Next, 25 μL of N-Methyl-N-(trimethylsilyl) trifluoroacetamide was added, and the mixture was incubated for 60 min at 45°C. Finally, a retention index standard mixture (*n*-alkanes) and an injection standard (4,4′-dibromooctafluorobiphenyl), both in hexane, were added to the mixture. The calibration consisted of six points for each quantified metabolite.

GC×GC–TOFMS experiment were carried out on an Agilent 7890 gas chromatograph equipped with a split/splitless injector (Agilent Technologies, Santa Clara, CA), cryogenic dual-stage modulator and time-of-flight mass spectrometer (Leco Corp., St. Joseph, MI, USA). In addition, multipurpose sampler with Maestro software (Gerstel, Mülheim an der Ruhr, Germany) was used for derivatization and sample introduction. A 10 m × 0.18 mm I.D. Rtx-5 (Restek Corp., Bellefonte, PA, USA) column with film thickness 0.20 μm was used as the first column and a 1.5 m × 0.1 mm I.D. BPX-50 (SGE Analytical Science, Austin, TX, USA) column with film thickness of 0.1 μm as the second column. A phenyl methyl deactivated retention gap column (1.5 m × 0.53 mm I.D.; Restek Corp., Bellefonte, PA, USA) was installed in front of the first column. The injector was used in the splitless mode at 250°C for injecting 1 μl of a sample. The splitless period was 90 s. High-purity helium (Yara Praxair AS, Oslo, Norway) was used as the carrier gas in a constant-pressure mode with initial pressure of 276 kPa. The first column oven temperature program was as follows: 50° (isothermal for 2 min) then 7°C/min to 240°C, and, finally, 25°/min to 300°C (3 min). The second dimension column oven temperature was maintained 15°C higher and the programming rate and hold times were similar than in the first dimension. The temperature of the transfer line was maintained at 260°C and ion source at 200°C. Modulation time was 4 s. Electron impact ionization was applied at 70 eV, and the mass range from 45 to 700 amu with 100 spectra/s were measured.

Next, the data files obtained by the ChromaTOF software were exported to text files and in-house developed software *Guineu* (Castillo et al., 2011) was used for aligning different data sets for further analyses. The original GC×GC–TOFMS data includes retention times, retention indices (*RI*), spectral information for possible identification, spectral similarity value (S = 0–999), and peak response data. The linear retention indices were calculated based on the total (i.e., sum of the first and the second dimension) retention times of the compounds and the retention times of the retention index standards (*n*-alkanes). The second dimension retention time is so short (1–3.5 s) that its contribution to the retention index is not significant. The alignment of the data was performed based on retention indices, second dimension retention times and spectra. Only quantified metabolites were included in the final dataset. The average relative standard deviation of the internal standard in the samples was 8.2%. The RSD for the quality control samples (pooled serum samples, *n* = 10) was on average 22%.

The GC×GC-TOFMS data acquired from a previous study (Oresic et al., 2011) of 37 AD patients and 46 controls were analyzed with the same method as in the present study. The internal standards were used for normalization of those data against the newly acquired data from NPH and BT patients.

### Statistical analysis

Statistical analyses were performed using METALAB R2014b (MathWorks, Natick, MA). The study groups were first compared using ANOVA. The data metabolites were log-transformed prior to the analysis. The False Discovery Rates (FDR) were calculated for the full dataset comprising metabolites (Storey, 2002). *Post-hoc* pairwise comparisons between the groups were performed using two-sided *t*-test using the Bonferroni correction.

## Results

Serum profiles of 21 metabolites were examined in 20 BT and 27 NPH patients. Clinical characteristics of the study subjects are shown in Table 1. Additionally, data from 37 AD patients and 46 HC from a previous study (Oresic et al., 2011) were analyzed together with the newly acquired data.

**Table 1:** Clinical characteristics of the NPH and Brain Tumor study subjects.

	**NPH**	**BT**	**AD**	**HC**
**DEMOGRAPHICS**				
N	26	20	37	46
Gender, male/female	15/11	15/5	17/20	21/25
Age (±SD)	73.7 ± 6.0	68.6 ± 6.2	74.2 ± 4.7	71.3 ± 6.3
MMSE Score (±SD)	24.5 ± 5.0	Not Collected	20.5 ± 2.9	25.8 ± 2.2
**BRAIN TUMOR TYPE**				
Grade 4 Glioblastoma, N (%)	N/A	17(85)	N/A	N/A
Grade 3 Anaplastic Oligodendroglioma, N (%)	N/A	2(10)	N/A	N/A
Grade 2 Oligodendroglioma, N (%)	N/A	1(5)	N/A	N/A

*AD, Alzheimer's disease; BT, brain tumor; HC, healthy controls; NPH, normal pressure hydrocephalus*.

**Figure 1:**
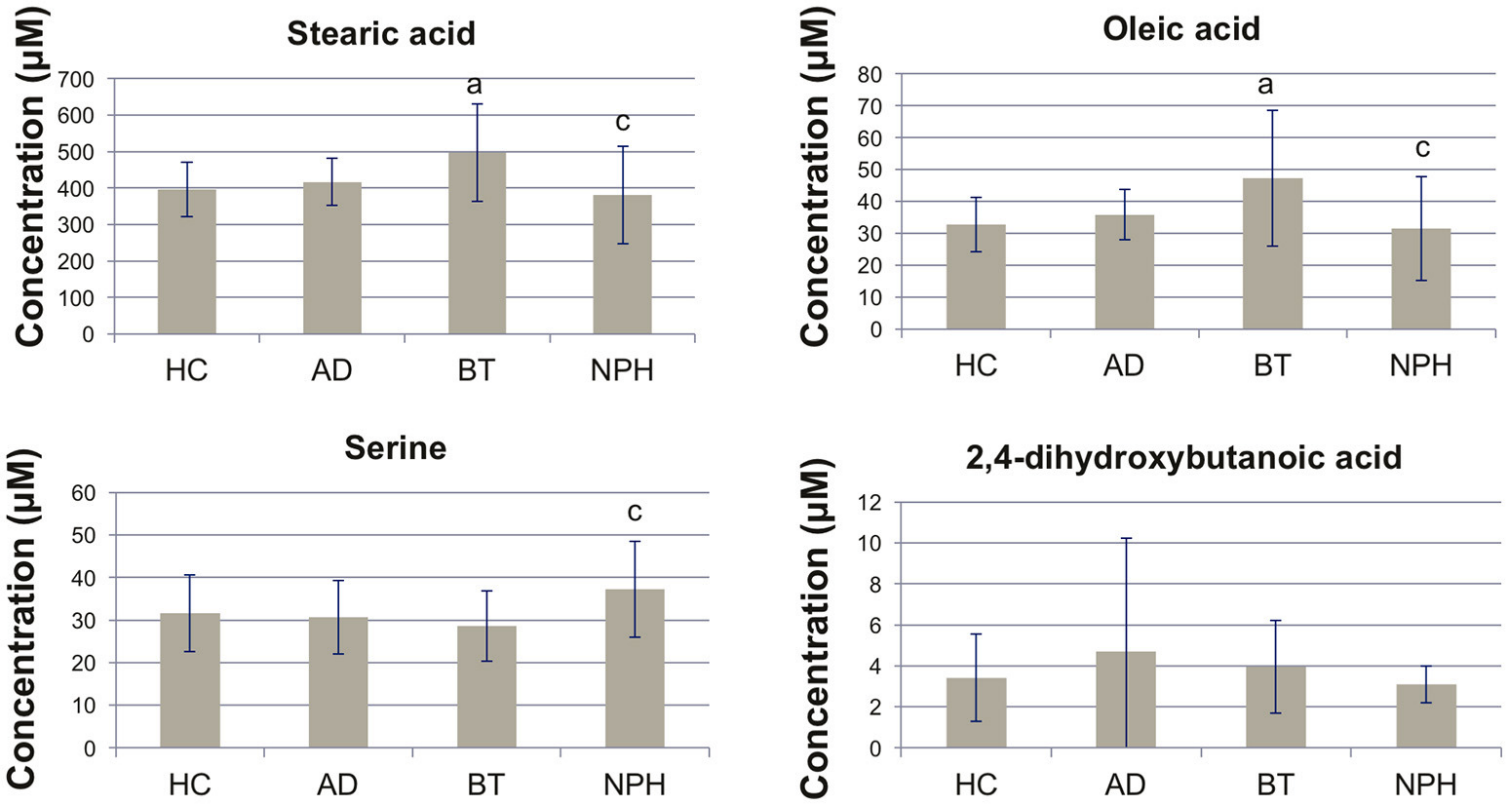
Bar plots showing concentrations of selected metabolites. Concentrations are shown as mean ± SD. Nominal *P* < 0.0167 (two-sided *t*-test) are abbreviated as follows in underlined columns: ^a^(HC vs. *AD*, HC vs. *BT* or HC vs. *NPH*), ^b^(AD vs. *BT* or AD vs. *NPH*), ^c^(BT vs. *NPH*). HC, healthy controls; AD, Alzheimer's disease; BT, brain tumor; NPH, normal pressure hydrocephalus.

We performed univariate analysis using analysis of variance (ANOVA), in order to quantitatively compare the metabolite profiles across the four study groups (Table 2). Ten (10) metabolites were significant following ANOVA analysis (FDR Q < 0.05) (Figure 1). 2,4-DHB showed no significant differences across the four groups, although the trend was that it was elevated in AD and decreased in NPH (Figure 1). Fatty acids were distinctly elevated in BT, while they were decreased in NPH as compared to BT but not as compared to HC except for linoleic acid (Figure 1, Table 2). The ketone body 3-hydroxybutyric acid was elevated in BT, while AD and NPH patients were also characterized by elevated glutamic acid, as well as lactic acid as compared to the BT group. Serine was specifically elevated in the NPH group (Figure 1).

**Table 2:** Metabolite concentrations across different study groups (μM), ±SD.

**Metabolite name**	***p*-value**	**HC**	**AD**	**BT**	**NPH**
Stearic acid	0.0003^*^	395.95 ± 74.83	416.60 ± 64.02	497.30 ± 133.52^a^	380.96 ± 133.99^c^
Oleic acid	0.0003^*^	32.75 ± 8.58	35.91 ± 7.94	47.28 ± 21.33^a^	31.49 ± 16.21^c^
Linoleic acid	0.3212	15.56 ± 2.79	15.71 ± 2.03	20.50 ± 9.03	13.54 ± 5.81^a^^,^^b^
Palmitic acid	0.0002^*^	93.89 ± 16.67	101.74 ± 22.12	122.70 ± 29.28^a^	95.99 ± 27.23^c^
Citric acid	0.0504^*^	22.68 ± 9.93	19.95 ± 7.35	18.58 ± 8.39	24.64 ± 10.60
Glutamic acid	0.0369^*^	10.05 ± 5.95	13.09 ± 6.80^a^	10.72 ± 4.22	12.81 ± 6.76
3-Hydroxybutyric acid	0.0307^*^	19.61 ± 23.12	13.01 ± 11.54	29.09 ± 33.44^b^	18.78 ± 13.70
2,4-dihydroxybutanoic acid	0.7404	34.29 ± 21.30	47.05 ± 55.41	39.63 ± 22.63	31.10 ± 8.99
Threonine	0.0407^*^	31.80 ± 10.18	31.63 ± 10.41	26.65 ± 7.13	35.39 ± 11.67^c^
Phenylalanine	0.8666	21.41 ± 7.73	22.97 ± 8.03	23.01 ± 10.61	22.64 ± 10.64
Serine	0.0278^*^	31.57 ± 8.97	30.70 ± 8.63	28.64 ± 8.23	37.26 ± 11.22^c^
Lactic acid	0.0210^*^	354.14 ± 109.76	403.70 ± 125.51	320.26 ± 137.24	390.39 ± 108.29
Methionine	0.7404	7.21 ± 2.77	7.46 ± 3.09	7.13 ± 1.63	7.59 ± 1.80
Glycine	0.2050	327.39 ± 126.63	349.69 ± 95.49	322.03 ± 110.43	376.68 ± 122.76
Isoleucine	0.8743	81.20 ± 29.51	81.86 ± 26.37	76.04 ± 29.64	85.49 ± 40.87
Leucine	0.7051	140.86 ± 53.35	141.68 ± 48.85	128.52 ± 48.38	147.08 ± 57.02
Valine	0.9686	335.79 ± 82.45	331.79 ± 84.95	330.35 ± 95.49	340.30 ± 92.07
Proline	0.5589	248.81 ± 95.98	252.63 ± 90.61	245.77 ± 209.66	233.13 ± 98.83
Cholesterol	0.1706	836.50 ± 209.61	847.78 ± 171.92	757.28 ± 219.13	886.58 ± 236.38
Alanine	0.0926^*^	257.78 ± 83.89	281.41 ± 129.80	231.94 ± 138.55	279.44 ± 94.27
Arachidonic acid	0.6901	21.27 ± 5.40	21.10 ± 4.78	21.37 ± 8.80	20.96 ± 9.94

* P-values are listed from the ANOVA analysis across all study group, and those with FDR q < 0.05 are marked with.

Post-hoc pairwise t-tests using the Bonferroni correction were performed across all pairs of study groups. Nominal P < 0.0167 are abbreviated as follows in underlined columns:

a (HC vs. AD, HC vs. BT or HC vs. NPH),

b (AD vs. BT or AD vs. NPH),

c *(BT vs. NPH). HC, healthy controls; AD, Alzheimer's disease; BT, brain tumor; NPH, normal pressure hydrocephalus*.

Gender differences in serum metabolite profiles have been reported in recent literature (Krumsiek et al., 2015). Since the gender distribution was not balanced across the study groups, we examined the effect of gender on metabolite profiles in HC group to assess the potential impact of gender imbalance on our results. Only three amino acids were diminished in females, leucine, valine and phenylalanine at a nominal *p* < 0.05. None of the metabolites displaying the differences across the study groups were affected by gender.

**Figure 2:**
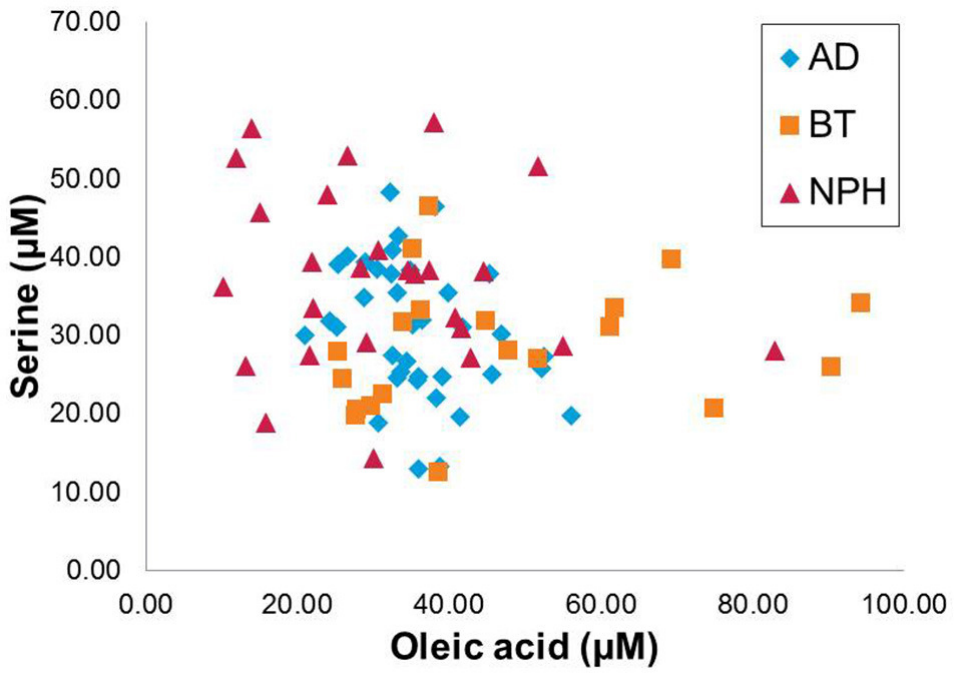
Scatter plot displaying the oleic acid and serine across the three CNS disease groups, Alzheimer's disease (AD), brain tumor (BT), and normal pressure hydrocephalus (NPH).

Figure 2 shows the scatter plot of concentrations of two selected discriminative metabolites: (a) oleic acid (which discriminates BT and NPH from other groups in opposite manner) and (b) serine (which discriminates NPH from the other groups, across the three disease groups). As can be concluded from the figure, AD, NPH, and BT have distinct metabolic signatures, with NPH and BT being the most different.

## Discussion

This study identifies metabolic profiles in three different CNS diseases, all known to be associated with hypoxia. No significant differences were observed between the levels of 2,4-DHB in BT or NPH as compared to HC. In an earlier study, we have shown that this metabolite is elevated during early progression of AD, while the changes are not significant in overt disease (there is however still trend toward the elevated levels as compared to HC) (Oresic et al., 2011). The results of the present study suggest that 2,4-DHB is not associated with hypoxia *per se*, because no changes were observed in BT and NPH, which are both linked to cerebral hypoxia (Zagzag et al., 2000; Dombrowski et al., 2008). Instead, 2,4-DHB may be associated with AD-specific metabolic derangements during the early phases of the disease. Recently, it has been hypothesized that production of 2,4-DHB in AD is linked to the hypoxia-induced activation of GABA shunt, which can be triggered by local hypoperfusion and subsequent hypoxia in AD brains caused by cerebral amyloid angiopathy (Salminen et al., 2016).

As a novel finding, the serum fatty acids were specifically elevated in BT. These changes may reflect lipid-related changes in tumor cells. *De novo* fatty acid synthesis is activated in tumors because this process is important for the biosynthesis of cellular membranes (Menendez and Lupu, 2007; Abramson, 2011). Glioblastomas are also known to contain higher levels of unsaturated fatty acids as compared to normal brain, which is indicative of exacerbated lipogenesis. Many genes regulating lipogenic pathways are upregulated in tumors, including SREBP-1 and its downstream-targeted genes acetyl-CoA carboxylase (ACC), fatty acid synthase (FAS) and low-density lipoprotein receptor (LDLR) (Gopal et al., 1963; Menendez and Lupu, 2007).

Serum serine was specifically increased in NPH as compared to BT. The importance of serine and its precursor glycine have been recognized in neurological and psychiatric disorders, although the present study is the first to report serine dysregulation in NPH (de Koning et al., 2003). Interestingly, glycine also tended to be upregulated in the present study, although the changes were not significant after correction for multiple comparisons. Serine is an important precursor of complex lipids, therefore its increased levels in NPH may reflect a compensatory response due to the diminished lipid levels, as reflected in decreased fatty acids in our study.

The ketone body 3-hydroxybutyric acid was elevated in BT. The tumor cells use glycolysis for energy and thus rely on glucose as the fuel. In contrast, neurons and glia can convert ketone bodies to fuel when glucose levels are reduced. Elevated levels of ketone bodies in BT as observed in our study may thus in part reflect decreased uptake of ketone bodies in the brain. It has been in fact hypothesized that dietary restriction together with ketogenic diet may be a strategy to manage cancer (Seyfried and Mukherjee, 2005). However, recent data from rats suggest that gliomas can oxidize ketone bodies when fed a ketogenic diet, thus contradicting this hypothesis (De Feyter et al., 2016). Ketone body supplementation and ketogenic diet have also been considered as a promising therapeutic approach in AD (Henderson et al., 2009; Krikorian et al., 2012). The ketones appear to reverse the amyloid-induced oxidation and disrupted function of a mitochondrial enzyme complex (Yin et al., 2016). In our study, 3-hydroxybutyric tended to be decreased in AD, but the observed change in mean values did not reach statistical significance in *post-hoc* analysis (*p* = 0.20, *t*-test HC vs. AD).

The human metabolome is affected by several factors including food intake. Since in our study the samples were taken in non-fasting state in two study groups (BT and NPH), this may have had an impact on the results, mainly by increasing variation of the metabolite levels. The impact of the post-prandial status varies between the metabolites. The concentrations of circulating free fatty acids in the circulation, for example, is dependent on the chain-length of the fatty acid. Free fatty acids with chain-length 16 carbons or more and glycerol are relatively stable immediately after the meal, and the levels are increased approximately 3 h after the meal, returning to the baseline 7–9 h later (Pellis et al., 2012). The same study has shown that most plasma amino acids increase directly after meal and then return to their baseline levels within 3–4 h. Particularly for the amino acids, the biological variation is however often larger than the effect of eating (Kim et al., 2014). In our study, the variability of fatty acid concentrations but not for other metabolites was clearly higher in the non-fasting BT and NPH groups. However, the mean values of their levels do not seem to have been affected, as they tend to be higher and lower, respectively, as in the HC group.

The main limitation of the present study is the fact that the metabolic profiling was acquired from two separate studies, performed in different study centers and at different time periods. In order to avoid any potential bias due to the well-known batch effect issues associated with comprehensive metabolomics studies in such circumstances (Dunn et al., 2011), we also limited our metabolic profiling approach only to metabolites that were quantified in both studies. The sample size was also too small to build reliable diagnostic models in cross-validation setting across the four study groups. However, even with such a limited sample size and coverage of metabolites, we were able to show the potential of using serum metabolomics as a tool to discriminate across multiple CNS disorders. Nevertheless, given the relatively small sample size, this study should be considered as preliminary and the findings need to be confirmed and expanded in the larger study setting.

In summary, our preliminary study suggests that three different CNS disorders, all characterized by hypoxia in specific stages of their pathogenesis, have different circulating metabolite signatures. This information, if confirmed in future studies, may help in the elucidation of disease-specific markers and thus also decrease the ambiguities as related to the disease diagnosis.

## Author contributions

MO and CB initiated the study. MO and GA wrote the first version of the manuscript. IM and TH performed metabolomics analysis. MO analyzed the data, with contributions from GA, MM, TH, and CB. GA, MM, and CB performed the clinical study in BT and NPH patients. HS coordinated the study in AD patients and controls. All authors reviewed and approved the manuscript.

### Conflict of interest statement

MO, TH, and HS are co-inventors in a patent application on metabolic markers of Alzheimer's disease. The other authors declare that the research was conducted in the absence of any commercial or financial relationships that could be construed as a potential conflict of interest.
